# A Test of the Adaptive Lag Hypothesis of the Evolution of Cancer Suppression and Lifespan in Dog Breeds

**DOI:** 10.3390/genes17020139

**Published:** 2026-01-27

**Authors:** Jack da Silva

**Affiliations:** School of Biological Sciences, University of Adelaide, Adelaide, SA 5005, Australia; jack.dasilva@adelaide.edu.au

**Keywords:** cancer, cancer suppression, Peto’s paradox, lifespan, dog, *Canis familiaris*

## Abstract

**Background/Objectives**: The well-established inverse relationship between lifespan and weight across dog breeds has been associated with higher cancer mortality in larger breeds. However, Peto’s paradox implies that larger-bodied species experience lower-than-expected rates of cancer mortality because of higher levels of cancer suppression. Therefore, it has been hypothesised that recently established large dog breeds experience high cancer mortality because of a lag in their evolution of cancer suppression. This “adaptive lag hypothesis” predicts that ancient breeds, which have had more time to evolve optimal cancer suppression, exhibit lower cancer mortality rates, longer lifespans, and smaller litter sizes (a cost of cancer suppression) compared to modern breeds of the same size. **Methods**: The adaptive lag hypothesis is tested here by comparing ancient and modern breeds defined by their levels of modern European genetic admixture. **Results**: Ancient breeds have significantly longer lifespans and smaller litters than modern breeds of the same size after controlling for phylogenetic relationships. The sparse data on cancer mortality rates of ancient breeds do not allow a definitive test of a difference between ancient and modern breeds, but ancient breeds show a significant departure from the increase in cancer mortality rate with weight observed for modern breeds. **Conclusions**: The results are consistent with the adaptive lag hypothesis, that the evolution of cancer suppression in large modern dog breeds has lagged behind their increased risk of cancer, thus shortening their lives compared to smaller breeds and compared to ancient breeds of the same size.

## 1. Introduction

The well-established decrease in individual lifespan with increasing body weight in dogs (*Canis familiaris*) has been associated with cancer mortality, the only studied cause of death whose rate increased with breed-specific body weight [1,2]. Larger individuals of any animal species have been expected to have greater susceptibility to cancer because most cancers are the result of somatic mutations arising from stem-cell division [3,4,5,6,7,8,9]. Taller humans also have on average shorter lives and higher rates of cancer [10,11,12]. The increase in cancer mortality rate with size in dogs may be explained by a multistage model of carcinogenesis with four driver mutations [13], which is consistent with an increase in the number of stem-cell divisions with size.

However, larger species of mammal generally live longer than smaller species [14,15,16,17]. In addition, larger species of mammal do not appear to have higher cancer mortality rates than smaller species [18,19,20,21,22], a pattern known as Peto’s paradox (but see [23]). Peto’s paradox implies that larger species invest more in cancer suppression [24,25,26], which appears to be the case [27,28,29]. More effective cancer suppression in larger species has also been consistent with only a 3-fold range in somatic mutation burden across mammals compared to a 40,000-fold range in body mass [30].

Peto’s paradox has led to the hypothesis that larger dog breeds experience higher rates of cancer mortality because the evolution of greater cancer suppression has lagged behind recent rapid increases in size [24,25,31]. Selection for extremely large size in some modern breeds has occurred very recently, well after breed establishment [32]. The hypothesis implicitly assumes that cancer suppression, through the inhibition and repair of DNA damage and the control of cellular proliferation [27,28], has fitness costs and thus is optimised to maximise fitness [33,34]. This assumption has been supported by reduced growth and reproduction in dogs and laboratory mice with increased cancer suppression [35,36,37,38,39,40,41,42]. Alternatively, there has been no selection for greater cancer suppression in large dog breeds because selective breeding typically occurred at younger ages, while most cancer mortality occurred at older ages [13]. The hypothesis that increased cancer suppression has lagged behind recent increases in size (the adaptive lag hypothesis) has been tested here with a recently published high-quality dataset on cancer mortality rates in dog breeds [43].

The adaptive lag hypothesis can be tested in dogs because most European breeds were established only in the last 200 years, while other “ancient” breeds were established more than 500 years ago [1,44,45,46,47,48,49]. The recent establishment of modern European breeds may have provided insufficient time for their evolution of optimal cancer suppression [1,43]. Thus, ancient breeds are predicted here to have evolved levels of cancer suppression closer to their optima than modern breeds. This hypothesis predicted that ancient breeds have lower cancer mortality rates, longer lifespans, and smaller litters (a cost of cancer suppression) than modern breeds of the same size.

To test the predictions of the adaptive lag hypothesis, published data on the genetic admixture of dog breeds [50,51] were used to identify ancient breeds and their lineal relationships. After controlling for phylogeny, ancient breeds were found to live longer and have smaller litters than modern breeds of the same size, as predicted. Cancer mortality rate increased with body size for modern breeds, but appeared to decrease with body size for the few ancient breeds for which data were available. These results were consistent with the adaptive lag hypothesis and could help explain the inverse relationship between lifespan and body weight in dogs.

## 2. Materials and Methods

### 2.1. Identification of Ancient and Modern Breeds

**Figure A1:**
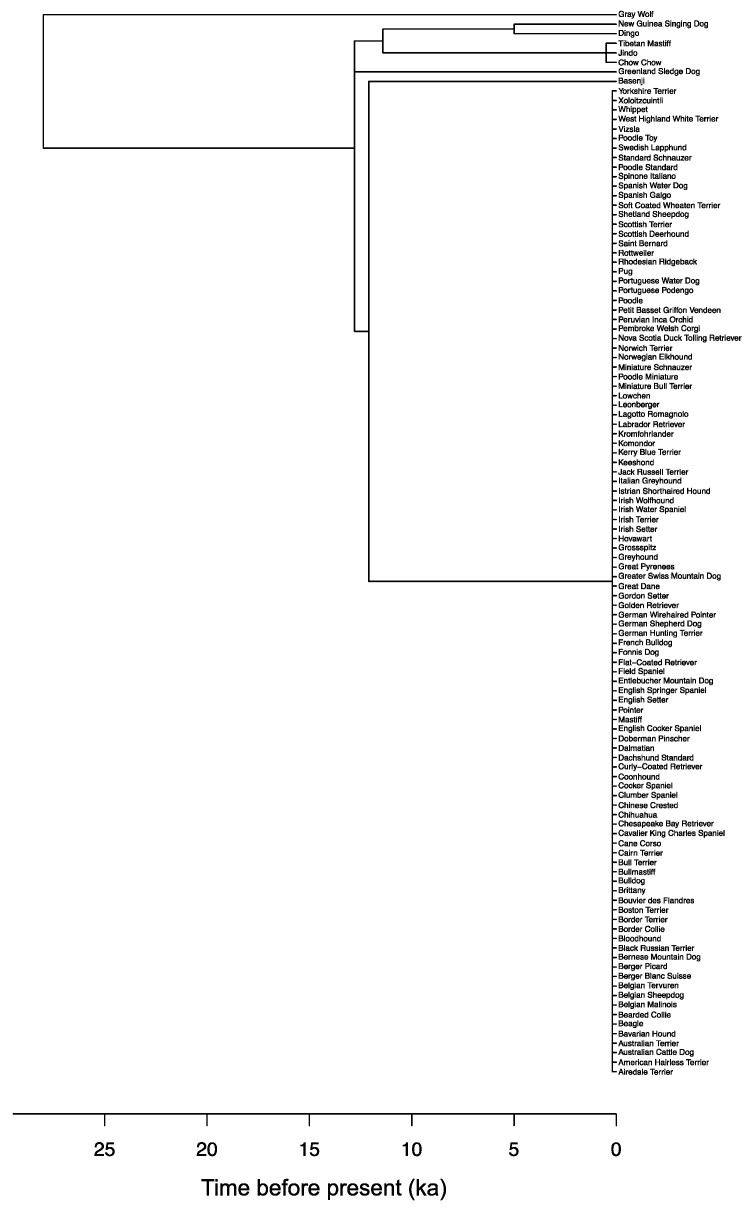
Phylogeny of dog breeds showing all ancient and modern breeds used in analyses. Scale is in thousands of years (ka).

The proportion of a breed’s modern European ancestry, based on the genetic admixture data [50], was the average of 10 individuals for each breed. Ancient breeds were defined as those containing <20% modern European ancestry. Seven ancient breeds were identified in this manner (Table A1). Using a less stringent cut-off resulted in the inclusion of breeds that were known to have been established recently (e.g., eurasier) [44]. Ancient breeds were compared to modern European breeds, defined as those containing ≥90% modern European ancestry. In total, 104 modern European breeds were identified (Figure A1).

### 2.2. Ancient Breed Lineages and Phylogeny

**Figure 1:**
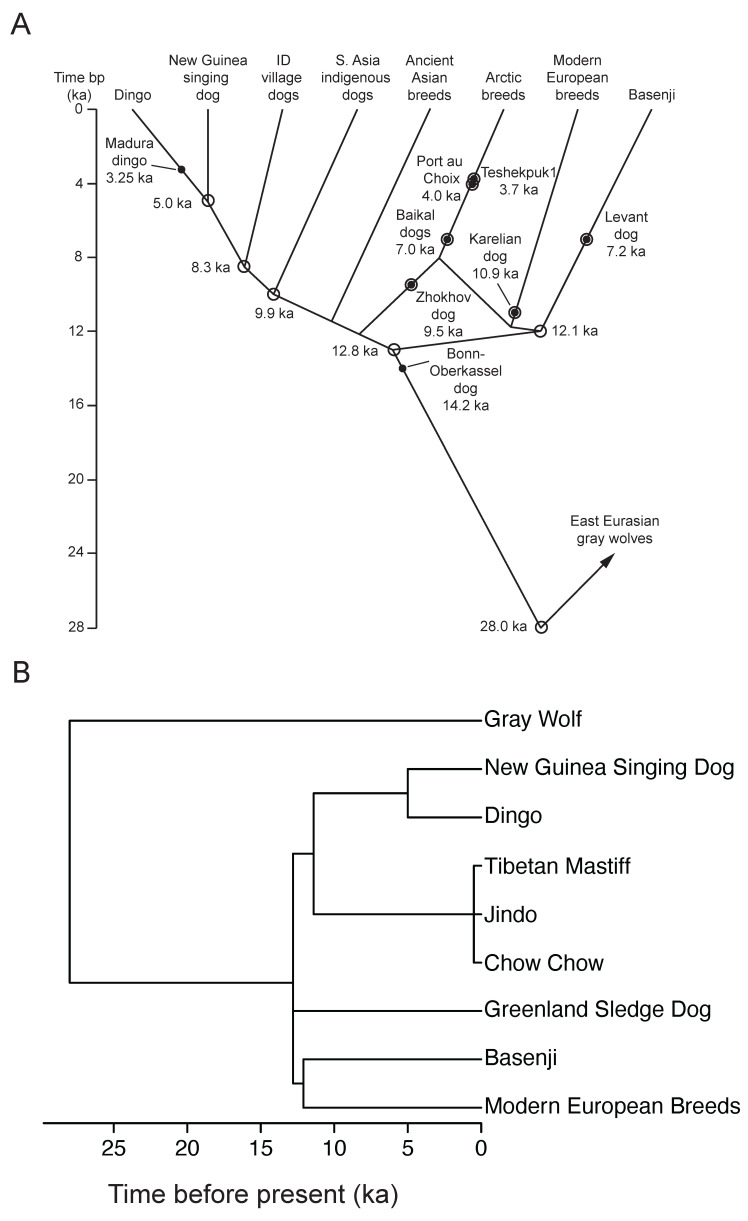
Ancient breed lineages. Time is in thousands of years (ka) before present (bp). (**A**) Composite diagram of lineage relationships of ancient dogs, including ancient admixtures. Dots indicate dates from archaeological/fossil evidence. Open circles indicate dates estimated from genetic data. Open circles with dots indicate dates from archaeological/fossil evidence and lineage placement based on ancient DNA admixture models or phylogenies. ID = Indonesia. (**B**) Phylogeny of ancient and modern breeds.

A composite diagram of the time-scaled lineage relationships of ancient dogs, including ancient admixtures (Figure 1A), was constructed from multiple sources [47,49,51,52,53,54,55]. This diagram was converted into a time-scaled phylogeny of breeds (Figure 1B) that was used in phylogenetic comparative analyses (see below). In constructing the breed phylogeny, the ancient Asian breeds (Tibetan mastiff, jindo, and chow chow) were assumed to form a polytomy with terminal branches of 500 years in length since their time-scaled phylogenetic relationships have not been determined [44,45,46]. The dates of origin for most modern European breeds in their current forms were not available, and those that were available were from the last 200 years [1,44]. Therefore, modern European breeds were represented as a polytomy with terminal branches of 200 years in length (Figure A1).

### 2.3. Breed Life History Data

High-quality data on body weights, lifespans, and causes of death for 118 breeds were from Kraus, Snyder-Mackler, and Promislow [43]. They reported breed-standard body weights primarily from the Fédération Cynologique Internationale. Their breed lifespans and causes of death were from the koiranet public database of the Finnish Kennel Club. Mean lifespans were calculated from owner-reported ages at death for breeds with at least 80 reported deaths in the database. Only the 1988–2002 birth cohorts were used to avoid underestimating lifespans due to the inclusion of incomplete birth cohorts. Dogs that died due to extrinsic causes were excluded to provide an estimate of the potential lifespan. The percent mortality due to cancer (cancer mortality rate) for each breed was calculated as the proportion of dogs that died of cancer out of the dogs with a diagnosed cause of death. Mean breed litter sizes were from 224 breeds registered in the Norwegian Kennel Club from 2006 to 2007 [56].

Data on body weight, lifespan, litter size, and cancer mortality rate were available from the above sources for only three ancient breeds: basenji, Tibetan mastiff, and chow chow. For the remaining ancient breeds (dingo, New Guinea singing dog, jindo, and Greenland sledge dog), weight, lifespan, and litter size data were from various sources (Table A1). For the dingo and New Guinea singing dog, which exist mainly as feral populations, these data were from captive individuals, providing the best comparisons to other breeds. These data were typically reported as a range of values, in which case the midpoint was used. Lifespans from these sources represent potential lifespans and were therefore comparable to the cohort-corrected lifespans from Kraus, Snyder-Mackler, and Promislow [43].

### 2.4. Phylogenetic Comparative Analyses

Phylogenetic generalised least squares (PGLS) analyses [57], as implemented by the R (version 4.4.1) package caper [58,59], were used to correct phylogenetic relationships [60]. Continuous variables were transformed when necessary to correct for curvilinearity or heteroscedasticity (non-homogeneity of variance), and to normalise the distribution of residuals to meet the assumptions of parametric analyses of variance [61] and the related PGLS analyses. Contrary to a recent claim [23], cancer mortality rate was expected to evolve like any other life history trait because it reflected a balance between susceptibility to cancer, dependent on body size, and resource allocation to cancer suppression, a form of somatic maintenance. Log transformation of cancer mortality rate as a proportion provided a better match to the assumptions than either the arcsine or logit transformations, which have been often recommended for analyses of proportions [62]. A test of departure from normality (Jarque–Bera test for normality in the R package moments) of phylogenetic residuals from a PGLS regression of log-transformed cancer mortality rate on log-transformed adult body mass showed no significant departure (*JB* = 0.053118, *p* = 0.9738).

## 3. Results

### 3.1. Lifespan

**Figure 2:**
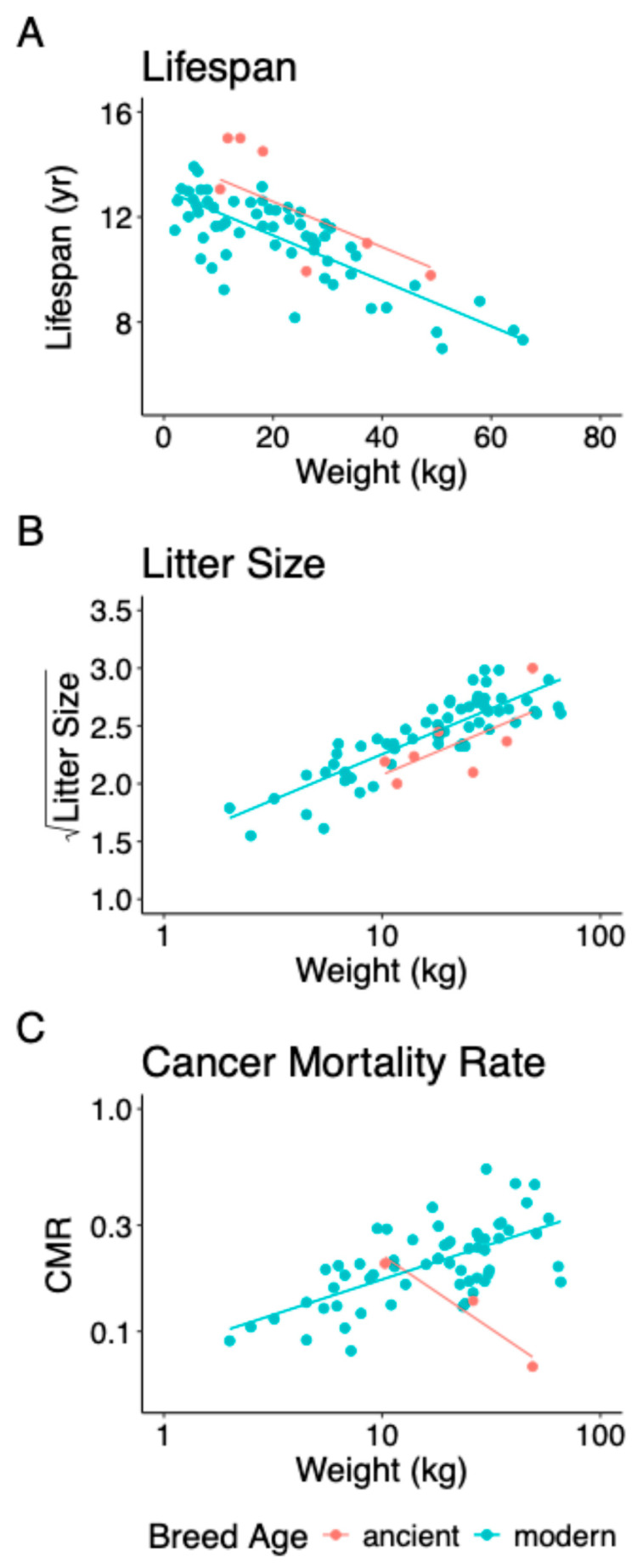
Phylogenetic generalised least squares analyses of covariance of the effect of breed age (ancient versus modern) with weight as a covariate. (**A**) Effect of breed age on lifespan: λ=0, κ=0.15, δ=3.0; $R^{2}=0.63,\;F_{2,65}=55.43,\;P=8.962\times 10^{-15}$. (**B**) Effect of breed age on litter size: λ=0, κ=0.49, δ=1.6; $R^{2}=0.72,\;F_{2,64}=80.38,\;P<2.2\times 10^{-16}$. (**C**) Effect of breed age on cancer mortality rate (CMR): λ=0; $R^{2}=0.48,\;F_{3,60}=18.47,\;P=1.328\times 10^{-8}$. Note that visual inspection of the distribution of data points is not a good guide to differences between breed ages because of the phylogenetic structure of breeds (all modern breeds have descended from a recent common ancestor).

Mean lifespan declined linearly with increasing body weight for both ancient and modern breeds, but ancient breeds had on average significantly longer lifespans across all body weights (no interaction) (Figure 2A; effect of breed age: ancient − modern = 1.2870 years (yr), SE = 0.3046, *t* = 4.2258, and *p* = 7.58 × 10^−5^). An increase of 10 kg predicted a decline of 0.87 years for both ancient and modern breeds (slope = −0.0865, SE = 0.0083, *t* = −10.3796, and *p* = 2.00 × 10^−15^), but ancient breeds lived 1.29 years longer.

A seemingly weak effect of phylogeny, reflected in the lack of phylogenetic signal in the residuals of the analysis (λ=0), was the result of all modern breeds analysed (104 breeds) emanating from a single recent node in the phylogeny, creating a polytomy (Figure 1B and Figure A1). When comparing modern and ancient breeds in the model, this phylogenetic structure was removed (running the analysis without breed age as a factor gave λ=1). However, there was evidence of recent rapid evolution of lifespan (δ=3) occurring mainly at breed divergences (κ=0.15), likely due to the indirect effects of recent strong selection on body size in modern breeds.

### 3.2. Litter Size

The square root of mean litter size increased linearly with the log of breed weight for both ancient and modern breeds, but ancient breeds had on average significantly smaller litters across all body weights (no interaction) (Figure 2B; effect of breed age: ancient − modern = −0.1778, SE = 0.0727, *t* = −2.4435, and *p* = 0.0173). Increasing weight from 10 kg to 50 kg, the range for the ancient breeds analysed, increases predicted litter size for ancient breeds from 4.32 to 6.92 and increases predicted litter size for modern breeds from 5.09 to 7.89.

### 3.3. Cancer Mortality Rate

Tests of the effects of breed age (ancient versus modern) on cancer mortality rate were limited by cancer mortality rate only being available for three ancient breeds: basenji, Tibetan mastiff, and chow chow. On log scales, cancer mortality rate increased linearly with weight for modern breeds (slope = 0.3187) but decreased for ancient breeds (slope = −0.6639) (Figure 2C; interaction effect: $F_{1,60}=11.9144,\;P=0.0010$). This result suggested that the largest ancient breeds invested more in cancer suppression. The result may seem surprising given so few data from ancient breeds, but the analysis accounted for the numbers of modern and ancient breeds and their phylogenetic relationships, including the fact that the modern breeds shared a very recent common ancestor. For modern breeds, increasing weight from 10 kg to 50 kg increased cancer mortality rate from 0.17 to 0.29.

The effects of each of lifespan and litter size on cancer mortality rate after controlling for weight and its interaction with breed age were also tested. Neither lifespan (slope = 0.0195, SE = 0.0145, *t* = 1.3448, and *p* = 0.1839) nor litter size (slope = 0.0287, SE = 0.0202, *t* = 1.4229, and *p* = 0.1601) explained any additional variance in cancer mortality rate.

## 4. Discussion

The increase in cancer mortality rate with body size in dogs was hypothesised to result from lags in the evolution of cancer suppression in response to the higher cancer burdens due to recent increases in size [24,25,31]. Assuming that ancient dog breeds have had more time than modern breeds to evolve cancer suppression optimal for their size, this hypothesis predicted that large ancient breeds would have longer lifespans, smaller litters, and lower cancer mortality rates than modern breeds of the same size. These predictions have been generally confirmed. Ancient breeds had on average longer lifespans and smaller litter sizes than modern European breeds of the same body weight. Unfortunately, the small number of ancient breeds for which cancer mortality rates were available prevented a firm conclusion being made about the difference in cancer mortality rates between modern and ancient breeds. However, while cancer mortality rate increased with weight for modern breeds, it appeared to decrease with weight for ancient breeds. This provided support for the hypothesis that ancient breeds have evolved greater cancer suppression at a cost of smaller litters. Such a trade-off was supported by findings that transgenic mice overexpressing the tumour suppressor gene *TP53* were not only more resistant to cancer, but also had lower growth rates, reduced size, shorter lifespans, and reduced fertility [40,41,42].

Alternatively, the longer lifespans and potentially lower cancer mortality rates of ancient breeds could be due to lower levels of inbreeding arising from less stringent selective breeding over longer periods, although there has been no evidence for this [13,43]. In addition, lower levels of inbreeding would not explain the smaller litter sizes (lower fertility) of ancient breeds.

Nunney [13] has analysed the relationships between cancer mortality rate and body mass and lifespan across dog breeds and concluded that the variation in cancer mortality rate was explained by a multi-stage model of carcinogenesis with four driver mutations. However, Nunney argued that there was no selection for increased cancer suppression in large breeds since selective breeding occurred at younger ages, while most cancer mortality occurred at older ages. Selective breeding tended to occur before age 7 yr [56], the mean lifespan of some giant breeds (Figure 2A), but there was substantial cancer mortality at these younger ages. The cancer mortality rate averaged across 82 breeds, each with ≥100 individuals sampled, increased linearly from 0.12 to 0.39 over the ages 2–7 yr, and peaked at 0.41 at 10 yr (Figure 3 in [2]). Therefore, at age 6 yr, the average cancer mortality rate was 0.34, 83% of the peak rate. Since these estimates were averages over a variety of breeds, giant breeds may be assumed to have even higher rates. In addition, although cancer mortality increased with age, the proportion of a birth cohort alive diminished with age and thus deaths at younger ages imposed stronger natural selection because younger individuals had more of their expected reproduction ahead of them [63,64]. Therefore, cancer mortality should impose significant selection on cancer suppression before age 7 yr in dogs.

This was investigated by compiling life tables for breed size classes using available data on probabilities of surviving from birth to age x ($l_{x}$) [65] and mean age-specific litter sizes [56]. Age-specific fecundity rate ($m_{x}$) was calculated from litter sizes assuming that 82.2% of females produced only one litter per year, 17.1% produced two litters, and 0.66% produced three litters [56]. From these data, fitness, measured as expected (mean) lifetime reproductive success, was calculated as $LRS=\sum_{x=0}^{\infty }l_{x}m_{x}$. Considering only reproduction spanning ages 2 to 6 yr (assuming $m_{x}=0$ at all other ages), the age range of consistent selective breeding [56], LRS for the giant breed size class (>45 kg) was 36.81 (Table 1). Then, if the mortality rate of a giant breed could be reduced through increased cancer suppression or reduced cancer susceptibility to that of a large breed (25–45 kg), increasing its life expectance at birth from 10.6 to 12.5 years [65] and decreasing its early-age litter sizes from ~7.5 to ~7.0 [56], a giant breed would increase its LRS by 2.84 offspring (Table 1). This gave a selection coefficient of s=2.84/36.81=0.08, indicating moderately strong selection. With a breed effective population size of $N_{e}=100$ [66], greater cancer suppression would be selectively favoured because $s>\left[1/\left(2N_{e}\right)=0.005\right]$ [67].

The longer lifespans and smaller litters of ancient breeds compared to modern breeds of the same size were consistent with the prediction that ancient breeds have evolved cancer suppression closer to their optima than modern breeds. The limited data on cancer mortality rate available for ancient breeds supported this conclusion in that the cancer mortality rate increased with weight for modern breeds but not for ancient breeds. Thus, the expectation that larger breeds were more susceptible to cancer mortality because of a greater number of stem cell divisions is supported. This increased risk of cancer mortality may then select for increased cancer suppression, depending on the age distribution of risk and the fitness cost of suppression. Analysis of the life table data for giant breeds showed that selection for increased cancer suppression or reduced cancer susceptibility at a cost of reduced reproduction was plausible. Therefore, considering that cancer is the only cause of death that increases with breed size [1,2], it appeared that the decrease in lifespan with breed size can be explained by increased susceptibility to cancer and a lag in the evolution of increased cancer suppression.

This study was unique in using genetic admixture data to identify ancient breeds in a quantitative manner. However, the lack of cancer mortality data for ancient breeds was a limitation of this study. Future work should focus on identifying additional ancient breeds and accurately measuring the cancer mortality rate of more ancient breeds.

## 5. Conclusions

It was hypothesised that the inverse relationship between lifespan and body size across dog breeds could be largely explained by the greater cancer susceptibility of modern large breeds and a lag in their evolution of optimal cancer suppression. This adaptive lag hypothesis predicted that ancient breeds lived longer, had smaller litters, and experienced lower rates of cancer mortality compared to modern breeds of the same size. It has been shown that ancient breeds had significantly longer lifespans and smaller litters than modern breeds of the same size after controlling for phylogenetic relationships. However, a lack of data prevented a definitive test of a difference in cancer mortality between ancient and modern breeds. Nevertheless, ancient breeds showed a significant departure from the increase in cancer mortality rate with weight observed for modern breeds. These results could offer tentative support for the adaptive lag hypothesis.

## Figures and Tables

**Table 1:** Life tables for breed size classes over ages of selective breeding ($m_{x}>0$).

	Large			Giant		
Age, *x* (yr)	*l_x_*	*m_x_*	*l_x_m_x_*	*l_x_*	*m_x_*	*l_x_m_x_*
2	0.9818	8.3	8.1358	0.9620	8.8	8.4272
3	0.9754	8.5	8.3137	0.9494	9.0	8.5416
4	0.9675	8.3	8.0173	0.9359	8.5	7.9770
5	0.9500	8.3	7.8723	0.8914	7.5	6.6480
6	0.9357	7.8	7.3107	0.8320	6.3	5.2201
LRS(Σ)			39.6497			36.8140

*l_x_*, probability of surviving from birth to age x, *m_x_*, fecundity at age x, and LRS, expected lifetime reproductive success.

## Data Availability

The original data presented in the study are openly available in Zenodo at https://doi.org/10.5281/zenodo.17983424.

## Abbreviations

The following abbreviations are used in this manuscript:

PGLS	Phylogenetic Generalised Least Squares
LRS	Lifetime Reproductive Success

## Appendix B

**Table A1:** Ancient dog breed data.

Breed	Modern European Admixture (%)	Mean Adult Mass (kg) *	Litter Size *	Midpoint Life Expectancy (yr) *
Basenji	15.3	10.43 [68]	4.8 [56]	13.5 [68]
Chow Chow	13.1	26.08 [68]	4.4 [56]	10 [68]
Dingo (captive)	7.0	14 [69]	5 [69]	15 [70]
Greenland Sledge Dog	17.0	37.25 [71]	5.6 [56]	11 [72]
Jindo	10.1	18.14 [68]	6 [73]	14.5 [68]
New Guinea Singing Dog (captive)	0.3	11.7 [69]	4 [74]	15 [75]
Tibetan Mastiff	5.1	48.76 [68]	9 [76]	11 [68]

* Sources of data are cited.
